# Population Structure of East African Relapsing Fever *Borrelia* spp.

**DOI:** 10.3201/eid1607.091085

**Published:** 2010-07

**Authors:** Sally J. Cutler, E. Margarita Bonilla, Rajbir J. Singh

**Affiliations:** Author affiliation: University of East London, London, UK

**Keywords:** Relapsing fever, Borrelia, louse-borne relapsing fever, tick-borne relapsing fever, vector-borne infections, Ethiopia, Tanzania, East Africa, bacteria, research

## Abstract

Differentiation of endemic East African tick-borne relapsing fever *Borrelia duttonii* spirochetes from epidemic louse-borne relapsing fever (LBRF) *B. recurrentis* spirochetes into different species has been questioned. We assessed a noncoding intragenic spacer (IGS) region to compare genotypes found in clinical samples from relapsing fever patients. Although IGS typing was highly discriminatory and resolved 4 East African tick-borne relapsing fever groups from a disease-endemic region in Tanzania, 2 IGS clades were found among LBRF patients in Ethiopia. The 2 IGS sequence types for *B. recurrentis* overlapped with 2 of the 4 groups found among *B. duttonii*. All cultivable isolates of *B. duttonii* fell into a single IGS cluster, which suggests their analysis might introduce selective bias. We provide further support that *B. recurrentis* is a subset of *B. duttonii* and represents an ecotype rather than a species. These observations have disease control implications and suggest LBRF *Borrelia* spp. could reemerge from its tick-borne reservoirs where vectors coexist.

## Abstract 2

*B. recurrentis* may have evolved directly from *B. duttonii*.

Relapsing fever is a recurrent febrile infection caused by various *Borrelia* spirochetes transmitted either by lice (epidemic relapsing fever) or by ticks (endemic relapsing fever). Relapsing fever was once a disease of global epidemic importance. However, largely as a result of the demise of the clothing louse *Pediculus humanus*, it is now restricted to areas where clothing lice are still commonplace, such as Ethiopia (*1*). In these regions it still has a major impact and was documented in a recent Ethiopian Department of Health report as being the seventh most common cause of hospital admission (2.5% of total; 3,777 cases) and fifth most common cause of death (0.9%, 42 cases) (2004) (*1*). The epidemic tendency of this infection is likely to reside in its vector transmission, with waves of clothing lice fleeing febrile patients and thus facilitating epidemic transmission. The endemic tick-borne relapsing fever spirochetes are transmitted by *Ornithodoros* ticks; *O. sonrai* serve as the principle vector for *Borrelia crocidurae* in West Africa, and *O. moubata* complex ticks effectively maintain these spirochetes in East Africa (*1*). Because soft ticks are only associated with hosts for their typically rapid nocturnal feeding, this limits the spread of infection beyond the confines of the areas where tick vectors reside.

Although these differing life styles of the vectors can account for the epidemiologic differences between these infections, recent studies have highlighted the similarity between *B. recurrentis*, the cause of louse-borne relapsing fever, and *B. duttonii*, the agent of East African of tick-borne relapsing fever (*2*–*4*). It has been postulated that *B. recurrentis* is a louse-adapted variant of *B. duttonii* (*5*). In these disease-endemic regions, diagnosis is typically achieved through demonstration of spirochetes in stained blood films from patients. However, this technique is unable to discriminate between the different *Borrelia* spp. that cause relapsing fever.

To provide a method able to reliably identify these spirochetes, we validated the use of sequence analysis of an intragenic spacer (IGS) region for typing these spirochetes (*3*). This method was used to analyze a noncoding spacer region and proved to be highly discriminatory; it resolved 4 groups among *B. duttonii* found among isolates and directly in tick vectors. Two groups were found among *B. recurrentis* isolates and louse vectors (*3*). Furthermore, a novel *Borrelia* species detected previously was found, and some sequence types resembled *B. crocidurae*, previously believed to be only in West Africa (*3*,*6*). The surprising finding was that 1 phylogenetic group of *B. duttonii* overlapped with a group of *B. recurrentis* (*3*). This colinearity was further supported by complete genomic sequencing of 1 isolate of *B. recurrentis* and 1 of *B. duttonii*, which suggests that *B. recurrentis* is a decaying genome that evolved from either *B. duttonii* or a common ancestral strain (*5*).

To further explore this apparent overlap between *B. duttonii* and *B. recurrentis*, we sequenced and compared additional gene targets. However, this study focused upon coding genes that were under different selective pressure and thus were not necessarily comparable to the noncoding IGS previously used. Furthermore, these investigations used only cultivable strains, and thus could represent bias toward those able to grow under axenic conditions. These investigations disclosed a clear demarcation between *B. duttonii* and *B. recurrentis* (*4*).

To resolve this apparent dichotomy, we undertook additional IGS typing directly on serum samples collected from patients with clinical cases, thus removing the selective pressure of cultivation. We report the results of those investigations.

## Materials and Methods

### Clinical Samples

Eighty-eight serum samples from patients in Ethiopia with relapsing fever that were blood film–positive for spirochetes were collected and stored frozen at –20°C before testing. Similarly, 23 samples collected from patients in Tanzania were collected and stored frozen. An additional series of 45 samples from family members accompanying patients to hospital as potential blood donors were available for analysis. Ethical approval for their collection and testing for relapsing fever had been granted for earlier studies (Ethiopia [*7*] and Commission for Science and Technology [COSTECH] 2001–330-NA-2001–25 for Tanzania).

### DNA Extraction

Serum samples were centrifuged at 13,000 rpm in a microfuge for 30 min and 100 μL of the pellet was used for DNA extraction. After an initial proteinase K digestion in a waterbath at 56°C for 1 h, DNA extraction was conducted by using DNeasy reagents (QIAGEN, Valencia, CA, USA) according to the manufacturer’s protocol for the QIAcube robotic platform with a final elution volume of 200 μL.

### Preliminary Screening for *Borrelia* spp.

A real-time TaqMan flagellin assay was used to screen samples for the presence of borrelial DNA before investigation. The primers are shown in Table 1. Primers were used at a final concentration of 1,000 mmol/L, and the probe was used at a final concentration of 50 mmol/L. A mastermix composed of PCR working concentration buffer without MgCl_2_ (Invitrogen, Carlsbad, CA, USA), 0.2 mmol/L of each dNTP, 5 mmol/L MgCl_2_, 0.075 nmol/L Rox, and 0.06 U Taq polymerase (Invitrogen). Mastermix was divided into aliquots of 23 μL to which 2 μL of extracted DNA was added. A MX3000 thermocycler (Stratagene, La Jolla, CA, USA) was used with an initial denaturation of 95°C for 10 min, followed by 50 cycles each at 94°C for 15 s and 60°C for 60 s. Results were read at a Fam wavelength (emission 516) by using Rox as a reference wavelength (emission 610).

**Table 1:** Primer and probe sequences used in study of East African relapsing fever *Borrelia* spp.*

Primer specificity	Primer/probe sequence, 5′ → 3′
Flagellin forward	CTAGTGGGCATAGAATTAATCGTGC
Flagellin reverse	GCTTGGGATAACCCTCTAATTTGA
Flagellin probe	fam-TGGTATGGGTGTTGCTGGGAAAATTACG-bhq1
First-round IGS forward	GTATGTTTAGTGAGGGGGGTG
First-round IGS reverse	GGATCATAGCTCAGGTGGTTAG
Second-round IGS forward	AGGGGGGTGAAGTCGTAACAAG
Second-round IGS reverse	GTCTGATAAACCTGAGGTCGGA

*IGS, intragenic spacer.

### IGS Typing

Samples positive for *Borrelia* DNA by the flagellin real-time assay were further subjected to IGS typing. A nested PCR was used to amplify the IGS, with outer primers anchored within the 3′ end of the *rrs* ribosomal gene and the 5′ end within the *ileT* gene as used previously (*3*). Primer sequences are shown in Table 1. Conventional PCR was performed by using 25-μL reaction volumes in My Cycler thermocylers (Bio-Rad, Hercules, CA, USA) with an initial denaturation at 94°C for 3 min, followed by 35 cycles at 94°C for 30 s, 55°C for 30 s, and 74°C for 60 s, and a final extension at 72°C for 7 min. A 2-μL volume from the first-round reaction was added to 23 μL of fresh second-round mastermix. This mixture was heated at 94°C for 3 min, followed by 35 cycles at 94°C for 30 s, 60°C for 30 s, and 74°C for 60 s, and a final extension at 72°C for 7 min. Resulting amplicons were resolved by electrophoresis on 1% agarose gels stained with ethidium bromide (0.5 μg/mL). Different manipulations of the PCR were conducted in different laboratory areas to avoid contamination, and nontemplate controls were included at a ratio of 1 for every 15 samples tested.

### Sequencing and Phylogenetic Analysis

Resulting amplicons were subjected to sequence analysis on an ABI 3700 automated DNA sequencer (Applied Biosystems, Foster City, CA, USA) at the Genome Centre Queen Mary’s University, London. The inner nested primers were additionally used for sequencing primers at a concentration of 10 pmol/μL. Sequence results were aligned by using ClustalW (www.ebi.ac.uk/clustalw) trimmed and a neighbor-joining phylogenetic tree was constructed by using MEGA4 (*8*).

## Results

Most samples collected from patients with either louse-borne (all 88 serum samples) or tick-borne relapsing fever (21/23 samples), yielded a positive PCR result upon initial screening and subsequently when tested for IGS. The 2 samples positive by initial microscopy, but negative by PCR, may represent DNA degradation over the prolonged period of frozen storage before testing. Additionally, 15 of 45 blood donors who accompanied patients in Tanzania had positive test results upon screening; samples from 9 of these also produced IGS amplicons. Nontemplate controls showed negative results.

IGS sequence data were obtained for 90% of the 124 PCR screen–positive samples (18 from patients with tick-borne relapsing fever, 85 from patients with louse-borne relapsing fever, and 9 from blood donors in Tanzania) and used for phylogenetic analysis. Only 5 of the 34 identical *B. recurrentis* type I sequences (represented by *B. recurrentis* A1 DQ000277) and 7 of the 43 *B. recurrentis* type II sequences (represented by *B. recurrentis* A11 DQ000278) are shown in Figure.

**Figure:**
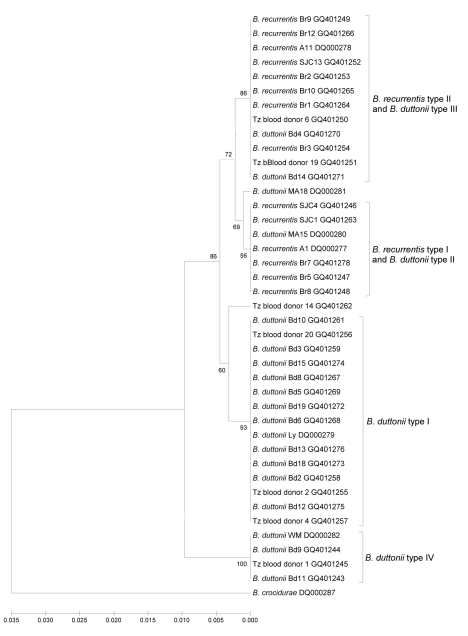
Phylogenetic tree of intragenic space sequences showing 2 groups among *Borrelia recurrentis* and 4 groups among *B. duttonii*. Tree created by using the unweighted pair group method with arithmetic mean. The *B. recurrentis* groups overlap with *B. duttonii* groups. Scale bar indicates nucleotide substitutions per site. Tz, Tanzania.

Similar to results found when looking at cultivable strains and IGS data in ticks or lice (*3*), we were able to show 4 clades among *B. duttonii* and 2 for *B. recurrentis* (Figure). Two of the *B. duttonii* clades contained only sequence types from patients in Tanzania or blood donors. Notably, all of the cultivable strains of *B. duttonii* resembled sequence type I. The other previously designated type IV (*3*), represented by *B. duttonii* WM (DQ000282) that had been represented by a single patient sequence, was found in 2 additional patient samples and in 1 serum sample from a blood donor. The remaining 2 *B. duttonii* clades overlapped with the 2 *B. recurrentis* clades. The accession numbers for the sequences types shown in Figure are deposited under GenBank nos. GQ401243–GQ401278 (Table 2). 

**Table 2:** Sample designation and accession numbers for *Borrelia recurrentis* and *B. duttonii,* East Africa

Strain designation	Species	GenBank accession no.	Reference
Bd11	*B. duttonii*	GQ401243	This study
Bd9	*B. duttonii*	GQ401244	This study
Tz blood donor 1	*B. duttonii*	GQ401245	This study
SJC4	*B. recurrentis*	GQ401246	This study
Br5	*B. recurrentis*	GQ401247	This study
Br8	*B, recurrentis*	GQ401248	This study
Br9	*B. recurrentis*	GQ401249	This study
Tz blood donor 6	*B. duttonii*	GQ401250	This study
Tz blood donor 19	*B. duttonii*	GQ401251	This study
SJC13	*B. recurrentis*	GQ401252	This study
Br2	*B. recurrentis*	GQ401253	This study
Br3	*B. recurrentis*	GQ401254	This study
Tz blood donor 2	*B. duttonii*	GQ401255	This study
Tz blood donor 20	*B. duttonii*	GQ401256	This study
Tz blood donor 4	*B. duttonii*	GQ401257	This study
Bd2	*B. duttoii*	GQ401258	This study
Bd3	*B. duttonii*	GQ401259	This study
Bd7	*B. duttonii*	GQ401260	This study
Bd10	*B. duttonii*	GQ401261	This study
Bd14	*B. duttonii*	GQ401262	This study
SJC1	*B. recurrentis*	GQ401263	This study
Br1	*B. recurrentis*	GQ401264	This study
Br10	*B. recurrentis*	GQ401265	This study
Br12	*B. recurrentis*	GQ401266	This study
Bd8	*B. duttonii*	GQ401267	This study
Bd6	*B. duttonii*	GQ401268	This study
Bd5	*B. duttonii*	GQ401269	This study
Bd4	*B. duttonii*	GQ401270	This study
Bd14	*B. duttonii*	GQ401271	This study
Bd19	*B. duttonii*	GQ401272	This study
Bd18	*B. duttonii*	GQ401273	This study
Bd15	*B. duttonii*	GQ401274	This study
Bd12	*B. duttonii*	GQ401275	This study
Bd13	*B. duttonii*	GQ401276	This study
Bd1	*B. duttonii*	GQ401277	This study
Br7	*B. recurrentis*	GQ401278	This study
A1	*B. recurrentis*	DQ000277	(*3*)
A11	*B. recurrentis*	DQ000278	(*3*)
Ly	*B. duttonii*	DQ000279	(*3*)
MA15	*B. duttonii*	DQ000280	(*3*)
MA18	*B. duttonii*	DQ000281	(*3*)
WM	*B. duttonii*	DQ000282	(*3*)

## Discussion

The findings of this study build upon those previously reported (*3*). As before, on the basis of IGS sequence types, we identified 4 distinct clusters of *B. duttonii*. Thus, the findings of this study, which analyzed sequence types in clinical samples, generally mirror results found when investigating sequence types of bacteria in arthropod vectors (*3*). The notable exception to this was the absence of the novel *Borrelia* sp. previously detected by Scott et al. in ticks (*3*) and by others in ticks and human samples (*6*,*9*). Whether detection of this species in humans with fever was directly related to the fever or incidental remains to be assessed through properly structured epidemiologic investigations. We did not detect this species among samples collected from patients with clinical relapsing fever in our study population. In consequence, our findings do not support the conclusion that this species has the potential to cause human infection.

Our samples from Tanzania were collected from villagers living in traditional mud-built dwellings; these dwellings usually have >80% infestation rates with *O. moubata* ticks. Regular nocturnal feeding by ticks could account for efficient transmission of these spirochetes to humans. Consequently, it is not surprising that samples from these persons, or from the asymptomatic blood donors who lived in similar conditions, were positive for *B. duttonii*. The absence of evidence of cross-contamination of the assays further substantiates these findings. Others have reported microscopy positive samples and PCR positive results from asymptomatic persons in Tanzania (*6*), which lends further support to this finding. Whether these observations represent persistent infection, as seen in the related spirochete that causes Lyme borreliosis, or reflects continued exposure by feeding ticks, remains to be elucidated.

Conversely, in Ethiopia, data are not available concerning the prevalence of soft tick species; however, infestation with clothing lice is commonplace (*10*). Based on the vector prevalence, in Ethiopia, relapsing fever has always been presumed to result from *B. recurrentis* infection. A subset of serum samples included in the current study were found to harbor a premature stop codon in their *recA* gene (S.J. Cutler et al., unpub. data), a feature disclosed in *B. recurrentis*, but not *B. duttonii,* in the recently completed whole genome sequencing of these spirochetes (*5*). This finding provides further evidence for the identification of infecting spirochetes among these samples.

The distribution of IGS profiles found among Tanzanian blood donors did not appear to differ from that found among persons with clinical cases in this region. The apparent absence of associated clinical signs is likely to correlate with a degree of local immunity. This finding is the basis of much local folk lore on the need to have regular tick exposure to boost immunity (*11*). These spirochetes undergo multiphasic antigenic variation (*12*), and whether they would continue to cause asymptomatic infection if the spirochetes underwent antigenic variation remains speculative. Furthermore, these organisms are able to evade key components of the innate immune response (*13*). This evasion might help account for their presence in asymptomatic persons.

Strikingly, the IGS sequence type for the cultivable isolates obtained for *B. duttonii* all fell into a single clade in which only *B. duttonii* sequence types were represented. This finding would explain the distinct clustering previously reported that separated *B. duttonii* from *B. recurrentis* when a multigene sequence–based approach was used (*4*). It may be that this particular cluster of *B. duttonii* is more readily cultivable that those belonging to other clades, thus highlighting the potential for misinterpretation of population structure analysis that is based only on cultivable isolates.

We had reported that *B. recurrentis* fell into 2 clades, which we can reconfirm with our current in situ findings (*3*). Of the samples tested from patients in Ethiopia with louse-borne relapsing fever, 44% fell into the type I clade and 56% were type II. Interestingly, our earlier work showed that *B. duttonii* type II represented by tick sample MA/15 (DQ000280) had a IGS sequence identical to those found for *B. recurrentis* type I from Ethiopia. Our current findings have provided further support for this observation by showing that another *B. duttonii* type III represented by tick sample MA/18 (DQ000281) aligns more closely with *B. recurrentis* IGS types. Further examples of IGS sequence homology were found between spirochetes that cause louse-borne relapsing fever and those that cause tick-borne relapsing fever, in samples from 2 patients from Tanzania with tick-borne relapsing fever (Bd4 and Bd14) and also from 2 blood donors from Tanzania (Tz blood donor 6 and Tz blood donor 19); the isolates clustered among *B. recurrentis* type II.

The vectorial specificity of these spirochetes has been a source of curiosity for many years. Early investigations questioned host and vector specificities of relapsing fever spirochetes; studies were carried out that enabled ticks to feed on patients with the louse-borne form of the disease and subsequently letting these ticks feed upon nonfebrile persons. These studies were unable to conclusively establish whether alternative arthropod vectors could transmit these spirochetes. Haberkorn demonstrated that *B. crocidurae* isolated from *O. erraticus* successfully multiplied in lice (*14*). The investigations by Heisch and Garnham initially claimed successful transmission of *B. duttonii* by body lice (*15*). However, in a later study Heisch concluded that *B. duttonii* from humans is unlikely to be naturally transmitted by lice (*16*). Many of these early investigations were hampered by the inability to reliably identify the organisms used, lack of cultivable strains, and having no means of assessing the immune status of recipient hosts. Speciation of the causative relapsing fever spirochete was based on the 1 vector–1 species criteria that relied on geographic region where infection occurred and identification of the vector responsible for transmission.

Collectively, these findings support the increasing evidence that *B. recurrentis* has evolved either directly from *B. duttonii* or from a common ancestral strain. This raises the question of whether these spirochetes should be considered as separate species or as ecotypes of the same species? With the advent of molecular typing tools, should the traditional classification of relapsing fever spirochetes be readdressed? A precedent for this approach already exists with other highly related organisms such as *Bacillus anthracis, B. cereus*, and *B. thuringiensis* (*17*). Although IGS typing provides a highly discriminatory tool to type these spirochetes, these observations should be underpinned by sequence analysis of multiple gene targets to provide a robust phylogenetic analysis.
